# CT-determined sarcopenia is associated with neutropenia in patients undergoing hyperthermic intraperitoneal chemotherapy for gastrointestinal cancer

**DOI:** 10.1186/s12957-023-02950-w

**Published:** 2023-02-22

**Authors:** Wei Jiang, Wenli Zhan, Fangxun He, Xiaolin Wu, Jing Wu, Xiangshang Xu, Zhixin Cao

**Affiliations:** Department of Gastrointestinal Surgery, Tongji Hospital of Tongji Medical College, Huazhong University of Science and Technology, No.1095 Jiefang Avenue, Wuhan, 430000 China

**Keywords:** Sarcopenia, Albumin, Leukopenia, Neutropenia, HIPEC

## Abstract

**Background:**

With better patient selection and the increasing experience in patients undergoing hyperthermic intraperitoneal chemotherapy (HIPEC) combined surgery, the rate of severe postoperative complications and mortality decreased significantly. However, leukopenia and neutropenia were still a particular concern, and their relation to sarcopenia was not clarified.

**Methods:**

Data of consecutive patients who underwent HIPEC for gastrointestinal cancer were collected and analyzed retrospectively between September 2020 and August 2022. Sarcopenia was assessed using psoas muscle index (PMI) at the L3 level on preoperative computed tomography (CT).

**Results:**

Among 103 patients enrolled, 37 (35.9%) were classified as sarcopenic. Most leukopenia and neutropenia occurred during the hospital leaving period after HIPEC and surgery. Before the first time of postoperative chemotherapy, the blood tests revealed 11 (29.73%) and 6 (9.09%) patients were diagnosed with neutropenia in sarcopenia and no sarcopenia groups, respectively. Logistic regression analysis revealed sarcopenia was independently associated with the increased risk of neutropenia (OR 5.58, 95% CI 1.70–18.29, *p* = 0.005). An incremental albumin level was protective against the occurrence of leukopenia and neutropenia.

**Conclusions:**

Sarcopenia and low albumin level were significantly associated with an increased rate of delayed neutropenia after HIPEC in that disease setting and could be the preoperative risk predictors.

**Supplementary Information:**

The online version contains supplementary material available at 10.1186/s12957-023-02950-w.

## Introduction

Peritoneal carcinoma (PC) is still considered a terminal condition for most patients with carcinomatosis and remains one of the most significant oncologic challenges [1]. PC was detected in more than 30% of patients with advanced gastric cancer and about 10–25% of patients with colorectal cancer [2–4]. Nowadays, it has been proven that the strategies variously combined systemic chemotherapy and hyperthermic intraperitoneal chemotherapy (HIPEC) with or without cytoreductive surgery (CRS) could improve survival and prevent morbid complications in the selected patients [5, 6].

Despite the encouraging results, HIPEC combined surgery for gastrointestinal cancer remained controversial because of the concern about its safety [5, 7]. Various studies focused on severe complications to identify prognostic factors in order to decrease morbidity [8–10]. With better patient selection and increasing experience, the rate of severe postoperative complications and mortality decreased significantly. However, despite the local administration of chemotherapy, leukopenia and neutropenia were still a particular concern in patients undergoing HIPEC [11, 12]. Delayed leukopenia or neutropenia after HIPEC was commonly observed in our clinical practice. They were associated with the risk of lethal infections as well as chemotherapy dose reductions and delays that may compromise treatment outcomes [13].

Sarcopenia, a key determinant of frailty and cancer cachexia, has been reported as a strong predictor of postoperative complications and prognosis across a wide range of gastrointestinal cancers [8, 14]. However, in patients undergoing CRS with HIPEC, the predictive function of sarcopenia remained controversial. And there were no studies that focus on the delayed leukopenia or neutropenia after HIPEC previously. This study focused on whether CT-determined sarcopenia was related to leukopenia or neutropenia after HIPEC for gastrointestinal cancers and tried to identify other parameters for predicting leukopenia or neutropenia.

## Materials and methods

### Patients and data collections

Data of consecutive patients who underwent HIPEC for gastrointestinal cancer in Tongji Hospital, Wuhan, China, were collected and analyzed retrospectively between September 2020 and August 2022. Patients who underwent prophylactic or therapeutic HIPEC were included in this study. Prophylactic HIPEC was defined as that HIPEC was performed after radical surgery for locally advanced gastrointestinal cancer with a high risk of peritoneal implantation to prevent a peritoneal recurrence. Therapeutic HIPEC was defined as that HIPEC was performed after CRS to treat peritoneal metastasis. According to our protocol, patients with Eastern Cooperative Oncology Group (ECOG) performance status score ≤ 1, resectable peritoneal metastases, and peritoneal cancer index ≤ 15 would undergo CRS combined HIPEC. Patients without computed tomography (CT) scan suitable for analysis or those who did not receive postoperative chemotherapy in our center were excluded for no recorded blood test.

This retrospective study was approved by the Ethics Committee of Tongji Hospital. Events occurring during hospitalization were recorded in our hospital’s database system. Data regarding general patient characteristics (e.g., age, sex, medical history), disease status (primary tumor location, pathological stage), treatment (surgical procedure, operative time, session number of HIPEC), and postoperative outcome (postoperative complications, mortality, length of hospital stay, and blood tests before first postoperative adjuvant chemotherapy) were reviewed. Comorbidity was defined as the patient diagnosed with at least one of these diseases: chronic lung disease, cardiovascular disease, cerebrovascular disease, diabetes, or another malignant tumor.

### Surgical and HIPEC procedure

Experienced surgical teams from Gastrointestinal Surgery Department performed radical or cytoreductive surgeries. All operations started with laparoscopic exploration to determine the suitable type of surgery. The radical surgeries included laparoscopic D2 gastrectomy, D3 colectomy, and total mesorectal excision (TME). During CRS, all macroscopically suspected peritoneal lesions would be surgically removed. Conversion to laparotomy would be performed if necessary. Before the abdominal wound was closed, four chemotherapeutic catheters (two inflow and two outflow tubes) were placed at the upper left, upper right, lower left, and lower right abdominal cavity, respectively.

According to the Chinese Expert Consensus on Clinical Application of HIPEC [15, 16] and the reaction of patients to HIPEC, one to three sessions of closed HIPEC were delivered within 7 days after surgery; the perfusion period for each session was 60–90 min. The interval between two adjacent sessions was 24–48 h. The chemotherapy drugs were paclitaxel (60 mg/m^2^) for gastric cancer and raltitrexed (3 mg/m^2^) with or without lobaplatin (50 mg/m^2^) for colorectal cancer; each dissolved in 3 l warmed nature saline at 43 ± 0.5℃ and then delivered into the abdominal cavity from an Automatic Hyperthermia Chemotherapy Perfusion Device (BR-TRG-II, Guangzhou, China) through the inflow tube placed under the diaphragm at a speed of 400 ml/min. Furthermore, the patients’ heart rate, blood pressure, respiration rate, and blood oxygen saturation values were monitored carefully. After the final session, two inflow and one outflow catheter would be removed; one outflow catheter remained as a drainage catheter for an additional 2 days.

After the discharge after surgery and HIPEC, the patient would be readmitted within 1 month for the first time of postoperative adjuvant chemotherapy. And before that, the general status and blood tests would be assessed.

### Postoperative complications

Leukopenia and neutropenia were defined according to the National Cancer Institute Common Terminology Criteria for Adverse Events, version 3.0, with leukopenia as a white blood count (WBC) lower than 3000 cell/ml^3^ and neutropenia as an absolute neutrophil count lower than 1500 cells/ml^3^ [17]. Granulocyte-colony-stimulating factor (G-CSF) would be therapeutically administered for leukopenia and neutropenia.

Postoperative complications were classified according to the Clavien-Dindo classification [18] of surgical complications. Severe postoperative complications were defined as those with a Clavien-Dindo classification of 3 or higher (e.g., anastomosis leakage, intensive care unit admission, or death.)

### Sarcopenia measurement

**Fig. 1:**
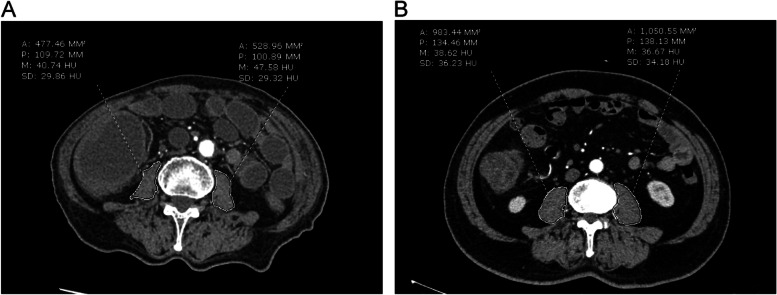
Measurement at the inferior end-plate level of the L3 vertebral body. Psoas (ROI) and the generated results were indicated by the dashed lines. **A** CT of a patient with sarcopenia; **B** CT of a patient without sarcopenia

Baseline weight and height were collected before surgery, calculating the body mass index (BMI) [BMI = weight (kg)/height squared (m^2^)]. An abdominal CT scan was performed as a part of the routine preoperative assessments. The image data were restored in the hospital’s imaging system (Synapse 3.2.1, Fujifilm Medical Systems, USA, Inc). Only preoperative abdominal CT scans were used. The measurement data were collected at the inferior end-plate level of the L3 vertebral body as the method verified by previous studies [19, 20]. The selected level should show an independent lumbar vertebral body area. Psoas was chosen as the measurement muscle for convenience. Use the selection tool of the imaging system to draw the region of interest (ROI), and the system would automatically generate the average CT value (HU) and area (mm^2^) (Fig. 1). Using this method, the psoas muscle index (PMI) was calculated by the formula as follows:

$$\mathrm{PMI}\;(\mathrm{mm}^2/\mathrm m^2)\:=\:(\mathrm{left}\;\mathrm{psoas}\;\mathrm{area}\;(\mathrm{mm}^2)\:+\:\mathrm{right}\;\mathrm{psoas}\;\mathrm{area}\;(\mathrm{mm}^2))/\mathrm{height}\;\mathrm{squared}\;(\mathrm m^2)$$

Two independent trained researchers completed the data collection and calculation without knowing the patients’ demographic and prognostic information. Before collecting image information, the two researchers assessed the quality in a consistent protocol of each image to decide whether to exclude the corresponding patient. The cut-off values of 545 mm^2^/m^2^ for men and 385 mm^2^/m^2^ for women defined by Jones [20] were used to classify patients as sarcopenic or nonsarcopenic.

### Statistical analysis

All statistical analyses were performed using IBM SPSS statistics version 22 (SPSS Inc., Chicago, IL, USA). Frequency data were presented as absolute numbers and percentages. Continuous data are presented as medians with interquartile ranges (IQRs) or means ± standard deviation (SD). Differences between groups were analyzed using the chi-square test for categorical variables and the Mann–Whitney *U* test or Student’s *t*-test for continuous variables, depending on normality tested by the Kolmogorov–Smirnov test. A two-tailed *p*-value lower than 0.05 was considered statistically significant. Odds ratios (ORs) with 95% confidence intervals (CIs) were calculated using logistic regression analysis to identify risk factors for the occurrence of leukopenia and neutropenia. Variables with a *p*-value lower than 0.1 resulting from the univariable logistic regression analysis were entered into the multivariable logistic regression analysis.

## Results

### Patients

Among 131 patients who had undergone prophylactic or therapeutic HIPEC, 103 (77.4%) had available assessable preoperative CT images and data of postoperative adjuvant chemotherapy. Because our center was a tertiary referral center, of those 28 patients excluded, 21 had a preoperative abdominal CT scan in local secondary hospitals; seven did not receive postoperative adjuvant chemotherapy in our hospital. The median age of these 103 patients was 56 (47, 63) years, and 63 (61.2%) of them were male. Forty (64.1%) patients had prophylactic HIPEC.

Depending on the referenced cut-off values, a total of 37 patients (35.9%) were classified as sarcopenic. Table 1 shows the comparison of baseline characteristics between the patients with sarcopenia and those without sarcopenia. The patients’ baseline and HIPEC characteristics between the two groups did not differ significantly.

**Table 1:** Patient baseline and HIPEC characteristics

Characteristic	Total (*n* = 103)	Sarcopenia (*n* = 37, 35.9%)	No sarcopenia (*n* = 66, 64.1%)	*p*
Gender				
Male	63	23	40	0.88
Female	40	14	26	
Age (years)	56 (47, 63)	57 (52, 63.50)	56 (45, 63.25)	0.40
BMI (kg/m^2^)	22.0 (19.5, 23.7)	22.5 (19.3, 24.0)	21.5 (19.5, 23.7)	0.57
Comorbidity	43	14	29	0.55
Tumor initial location				
Stomach	67	23	44	0.65
Colorectum	36	14	22	
Preoperative chemotherapy	19	7	12	0.93
Preoperative test results				
Albumin (g/l)	38.20 (36.03, 40.98)	39.2 (35.85, 41.3)	38.2 (35.70, 41.3)	0.53
WBC (× 10^9^/l)	5.3 (4.45, 6.53)	5.73 (4.59, 6.77)	5.14 (4.40, 6.37)	0.45
Neutrophil (× 10^9^/l)	3.21 (2.52, 4.07)	3.49 (2.83, 4.06)	2.95 (2.46, 4.13)	0.28
Type of HIPEC				
Prophylactic	66	26	40	0.33
Therapeutic	37	11	26	
Session of HIPEC				
One	13	4	9	0.90
Two	37	14	23	
Three	53	19	34	

*BMI* body mass index, *WBC* white blood count, *HIPEC* hyperthermic intraperitoneal chemotherapy

Since the TNM stages were different between gastric and colorectal cancer, we also compare the TNM stages between sarcopenia and no sarcopenia groups based on cancer types. The differences did not show any statistical significance (Tables S1 and S2).

The preoperative albumin values of gastric and colorectal cancer were 38.2 ± 4.4 g/l and 38.7 ± 2.8 g/l (*p* = 0.46), respectively. The albumin values of gastric and colorectal cancer before the discharge were 32.6 ± 3.8 g/l and 32.5 ± 3.6 g/l (*p* = 0.92), respectively.

### Leukopenia, neutropenia, and postoperative outcomes

On the seventh day after surgery, leukopenia was observed in one and five patients in sarcopenia and no sarcopenia groups, respectively, and neutropenia was observed in two patients in the no sarcopenia group. Before the first time of postoperative chemotherapy, the blood tests revealed 10 (27.0%) and 9 (13.6%) patients with leukopenia in sarcopenia and no sarcopenia groups, respectively, and 11 (29.7%) and 4 (9.1%) patients were diagnosed with neutropenia in sarcopenia and no sarcopenia groups, respectively. Sarcopenia was significantly associated with the occurrence of neutropenia (*p*-value = 0.007); the patients with sarcopenia likely had a higher rate of leukopenia. However, the difference was not significant (*p*-value = 0.093) (Table 2).

**Table 2:** Comparison of PMI and outcomes between two groups

Characteristic	Sarcopenia (*n* = 37, 35.9%)	No sarcopenia (*n* = 66, 64.1%)	*p*
CT-to-surgery interval (days)	6 (5, 9.5)	5 (3, 9)	0.24
PMI (mm^2^/m^2^)			
Male	440.37 (387.74, 505.34)	658.32 (601.86, 760.76)	< 0.001^**^
Female	308.90 (253.53, 339.47)	489.01 (446.94, 611.14)	< 0.001^**^
Abdominal complications	3	5	0.16
Postoperative stay (days)	10 (8, 11)	9 (8, 10.25)	0.16
Leukopenia			
7 days after surgery	1	5	0.42
Before first postoperative chemotherapy	10	9	0.093
Neutropenia			
7 days after surgery	0	2	0.54
Before first postoperative chemotherapy	11	6	0.007^*^

*PMI* psoas muscle index

^*^p < 0.05; ^**^p < 0.01

Of 67 patients with gastric cancer, 12 and 15 patients were diagnosed with neutropenia and leukopenia, respectively; of 36 patients with colorectal cancer, 5 and 4 patients were diagnosed with neutropenia and leukopenia, respectively. However, the differences did not show statistical significance (*p* = 0.60 for neutropenia and *p* = 0.16 for leukopenia).

No severe postoperative complications were recorded. Among the patients with sarcopenia, three abdominal complications occurred: one patient with a chyle leak and two patients with prolonged postoperative ileus. Additionally, in the no sarcopenia group, one patient was found with intra-abdominal infection and four with prolonged postoperative ileus. The incidence rates of complications did not differ significantly between the two groups.

### Multivariate analysis

In the univariate logistic regression analysis of leukopenia, only preoperative serum albumin level (OR 0.86, 95% CI 0.75–0.98, *p* = 0.027) reached statistical significance, and two variables (sarcopenia and albumin) with a *p*-value lower than 0.1 were included in the multivariate logistic regression model. Preoperative albumin level (OR 0.82, 95% CI 0.69–0.99, *p* = 0.04) was independently associated with the risk of leukopenia. Sarcopenia was not an independent risk factor statistically (OR 2.76, 95% CI 0.96–7.96, *p* = 0.06) (Table 3).

**Table 3:** Logistic regression analysis for risk factors associated with leukopenia before first postoperative chemotherapy

	Univariable OR (95% CI)	*p*	Multivariable OR (95% CI)	*p*
Sarcopenia	2.35 (0.85, 6.44)	0.098	2.76 (0.96, 7.96)	0.06
Gender				
Female	1			
Male	1.11 (0.40, 3.11)	0.84		
Age (years)	1.00 (0.96, 1.04)	0.88		
BMI (kg/m^2^)	1.02 (0.88, 1.19)	0.79		
Comorbidity	0.59 (0.20, 1.69)	0.32		
Tumor initial location				
Stomach	1			
Colorectum	0.43 (0.13, 1.42)	0.17		
Preoperative chemotherapy	1.2 (0.36, 4.22)	0.75		
Preoperative test results				
Albumin (g/l)	0.86 (0.75, 0.98)	0.027^*^	0.85 (0.73, 0.97)	0.018^*^
WBC (× 10^9^/l)	0.82 (0.61, 1.11)	0.20		
Neutrophil (× 10^9^/l)	0.81 (0.55, 1.18)	0.26		
Type of HIPEC				
Prophylactic	1			
Therapeutic	1.1 (0.37, 2.95)	0.93		
Session of HIPEC				
One	1			
Two	1.47 (0.34, 6.42)	0.61		
Three	1.14 (0.38, 3.40)	0.81		

*BMI* body mass index, *WBC* white blood count, *HIPEC* hyperthermic intraperitoneal chemotherapy

A *p*-value lower than 0.1 resulting from the univariable logistic regression analysis was entered into the multivariable logistic regression analysis

^*^p < 0.05

In the univariate logistic regression analysis regarding neutropenia, sarcopenia (OR 4.23, 95% CI 1.41–12.66, *p* = 0.01) and preoperative albumin level (OR 0.84, 95% CI 0.72–0.97,* p* = 0.015) also reached statistical significance and were included in the multivariate logistic regression model. Sarcopenia (OR 5.58, 95% CI 1.70–18.29, *p* = 0.005) and preoperative albumin level (OR 0.81, 95% CI 0.69–0.95, *p* = 0.008) were also independently associated with neutropenia occurrence (Table 4).

**Table 4:** Logistic regression analysis for risk factors associated with neutropenia before first postoperative chemotherapy

	Univariable OR (95% CI)	*p*	Multivariable OR (95% CI)	*p*
Sarcopenia	4.23 (1.41, 12.66)	0.01^*^	5.58 (1.70, 18.29)	0.005^*^
Gender				
Female	1			
Male	0.89 (0.31, 2.57)	0.83		
Age (years)	0.99 (0.95, 1.03)	0.45		
BMI (kg/m^2^)	0.98 (0.83, 1.16)	0.82		
Comorbidity	0.72 (0.25, 2.13)	0.56		
Tumor initial location				
Stomach	1			
Colorectum	0.74 (0.24, 2.29)	0.60		
Preoperative chemotherapy	2.14 (0.65, 7.04)	0.21		
Preoperative tests results				
Albumin (g/l)	0.84 (0.72, 0.97)	0.015^*^	0.81 (0.69, 0.95)	0.008^*^
WBC (× 10^9^/l)	0.90 (0.67, 1.21)	0.48		
Neutrophil (× 10^9^/l)	0.77 (0.51, 1.16)	0.21		
Type of HIPEC				
Prophylactic	1			
Therapeutic	0.70 (0.23, 2.18)	0.54		
Session of HIPEC				
One	1			
Two	0.67 (0.11, 4.15)	0.66		
Three	1.44 (0.28, 7.47)	0.66		

*BMI* body mass index, *WBC* white blood count, *HIPEC* hyperthermic intraperitoneal chemotherapy

A *p*-value lower than 0.1 resulting from the univariable logistic regression analysis was entered into the multivariable logistic regression analysis

^*^p < 0.05

## Discussion

Leukopenia and neutropenia related to HIPEC toxicity increase the risk of lethal infections as well as chemotherapy dose reductions and delays [13]. Predictive factors are pivotal to improving the treatment strategies to improve survival. Our study proved that CT-determined sarcopenia and low serum albumin level were independently associated with an increased rate of leukopenia and neutropenia after HIPEC. According to our results, new strategies with G-CFS and nutrition support should be established for the patients with these risk factors.

In this study, the incidences of leukopenia and neutropenia before the first time of the postoperative adjuvant chemotherapy were 18.4% and 16.5%, respectively. Most of them occurred during the leaving hospital period after HIPEC and surgery. Despite the incidences in the range reported by previous studies **[**11, 15, 21], the onset times were a little later than in these studies. Additionally, our study’s severe postoperative complication rate was lower than other reports. These differences might attribute to the different study populations and HIPEC procedures. The inclusion criteria that the patients needed the blood test results before postoperative adjuvant chemotherapy might exclude some patients with severe complications. Moreover, the majority of HIPEC was prophylactic type in our study would lead to a relatively more minor extent of the operation. What is more, unlike most other studies, the closed HIPEC of our research was delivered within 7 days after surgery instead of during surgery. And the sessions of HIPEC were determined according to the reaction of patients, which would help avoid some severe complications.

Since first described by H. Rosenberg [22] in 1988, sarcopenia, which is used to describe the age-related loss of skeletal muscle quantity and quality, has received increasing attention. The quantity and quality of muscles could be based on CT or magnetic resonance imaging (MRI) as the gold standard [23–26]. And in practical applications, imaging was used as a routine examination item for diagnosis to evaluate the state of skeletal muscles. In our study, the incidence of sarcopenia according to CT scan was 48.53%, similar to other studies [8, 21]. Many studies defined the cut-off value as the lowest quartile or optimum stratification of sex-specific CT measurements [14]. Nevertheless, patients with relatively later-stage cancer are more likely to develop muscle depletion because of consumption, metabolic change, and systemic inflammation [27]. And our study had a limited number of cases, leading to inaccurate cut-off values if determined by statistical method. So, we chose the cut-off value reported by previous studies. The optimal and universal cut-off value of CT measurements might need to determine by an epidemiological study with a large sample size in the future.

Sarcopenia has been reported by a few studies [28–30] as a predictive factor of hematologic toxicity or neutropenia in widely oncology populations. However, previously, only one study revealed sarcopenia was independently associated with hematologic toxicity linked to HIPEC alone in patients with colorectal cancer [21]. And in that study, only grades III or IV neutropenia was considered as events. Our study on a gastrointestinal cancer population revealed that the association between sarcopenia and leukopenia or neutropenia was universal in patients undergoing HIPEC. For the patients with sarcopenia undergoing HIPEC, even without severe postoperative complications and being discharged smoothly, monitoring leukopenia and neutropenia is essential during the period of leaving the hospital. Then, for those diagnosed with leukopenia and neutropenia during that period, G-CFS could be administrated in advance before the first postoperative chemotherapy.

Serum albumin was traditionally considered as a marker reflecting nutritional status and included in the nutrition support enhancement pathway. Various studies have revealed that the nutrition status and serum albumin were related to systemic chemotherapy-induced hematological toxicities [13, 31, 32]. Previous studies in the population undergoing CRS plus HIPEC suggested that the albumin level was associated with overall survival [33, 34]. Some other studies showed that low serum albumin would increase mortality and postoperative complications [35, 36]. Our results showed that an incremental albumin level was protective against the occurrence of leukopenia and neutropenia after HIPEC. Thus, for patients with hypoalbuminemia undergoing HIPEC, preoperative nutrition support with monitoring of albumin level should be necessary.

There were some limitations of our study. First, it was a retrospective cohort study, leading to some selection bias. Second, as mentioned before, the inclusion criteria might result in the additional exclusion of the patients with severe complications and those transferred to other centers for personal reasons. There was a certain degree of data loss. Third, our study had relatively small population samples and heterogeneous managements, which might affect the results. Finally, we did not evaluate the long-term clinical outcome in this study.

## Conclusions

This study showed that sarcopenia and low albumin level were significantly associated with an increased rate of neutropenia in patients undergoing HIPEC combined surgery for gastrointestinal cancers and could be used in preoperative risk prediction. The treatment strategies should be optimized for the patients with these risk factors.

## Supplementary Information

**Additional file 1: Table S1.** Comparison of the TNM stages between Sarcopenia and No Sarcopenia groups in patients with gastric cancer. **Table S2.** Comparison of the TNM stages between Sarcopenia and No Sarcopenia groups in patients with colorectal cancer.

## Data Availability

All data are available from the corresponding authors.

## Abbreviations

HIPEC: Hyperthermic intraperitoneal chemotherapy
CT: Computed tomography
PMI: Psoas muscle index
PC: Peritoneal carcinoma
CRS: Cytoreductive surgery
ECOG: Eastern Cooperative Oncology Group
TME: Total mesorectal excision
BMI: Body mass index
WBC: White blood count
G-CSF: Granulocyte-colony-stimulating factor
ROI: Region of interest
IQRs: Interquartile ranges
ORs: Odds ratios
CIs: Confidence intervals
MRI: Magnetic resonance imaging
