# Supplementary material for: CT-determined sarcopenia is associated with neutropenia in patients undergoing hyperthermic intraperitoneal chemotherapy for gastrointestinal cancer
Source: World J Surg Oncol. 2023 Feb 22;21:57. doi: 10.1186/s12957-023-02950-w (PMC9945613; doi:10.1186/s12957-023-02950-w)
Supplement: Supplementary file 1 — Additional file 1: Table S1. Comparison of the TNM stages between Sarcopenia and No Sarcopenia groups in patients with gastric cancer. Table S2. Comparison of the TNM stages between Sarcopenia and No Sarcopenia groups in patients with colorectal cancer. [file 12957_2023_2950_MOESM1_ESM.doc]

Table S1. Comparison of the TNM stages between Sarcopenia and No Sarcopenia groups in patients with gastric cancer.

| TNM stage | | Sarcopenia  (n=23) | No Sarcopenia  (n=44) | P value |
| --- | --- | --- | --- | --- |
| T | |  |  | 0.96 |
|  | 3 | 8 | 15 |  |
|  | 4 | 15 | 29 |  |
| N | |  |  | 0.84 |
|  | 0 | 2 | 5 |  |
|  | 1 | 5 | 7 |  |
|  | 2 | 4 | 11 |  |
|  | 3 | 12 | 21 |  |
| M |  |  |  | 0.19 |
|  | 0 | 20 | 32 |  |
|  | 1 | 3 | 12 |  |

Table S2. Comparison of the TNM stages between Sarcopenia and No Sarcopenia groups in patients with colorectal cancer.

| TNM stage | | Sarcopenia  (n=14) | No Sarcopenia  (n=22) | P value |
| --- | --- | --- | --- | --- |
| T | |  |  | 0.59 |
|  | 3 | 5 | 6 |  |
|  | 4 | 9 | 16 |  |
| N | |  |  | 0.21 |
|  | 0 | 2 | 7 |  |
|  | 1 | 7 | 5 |  |
|  | 2 | 5 | 10 |  |
| M |  |  |  | 0.56 |
|  | 0 | 5 | 10 |  |
|  | 1 | 9 | 12 |  |
